# Supplementary material for: Proteomics Reveals the Potential Protective Mechanism of Hydrogen Sulfide on Retinal Ganglion Cells in an Ischemia/Reperfusion Injury Animal Model
Source: Pharmaceuticals (Basel). 2020 Aug 27;13(9):213. doi: 10.3390/ph13090213 (PMC7557839; doi:10.3390/ph13090213)
Supplement: Supplementary file 1 [file pharmaceuticals-13-00213-s001.zip › pharmaceuticals-897815-sup/#Supplementary data/Supplementry data_3_Mass Spectrometry Sample Preparation.docx]

## Mass Spectrometry Sample Preparation

### Protein extraction

The rest of retinal explants were rinsed in ice-cold PBS to remove contaminants and weighed immediately after. Protein extraction from the retinal samples was carried out using T-PER Tissue Protein Extraction Reagent(Thermo Scientific Inc., Waltham, MA, USA) following an in-house established method catered especially for small amounts of samples, as described by Manicam et al.[[24](#_ENREF_24)] Briefly, 400 μl T-PER reagent was added to each retina, subsequently, retinal samples were subjected to homogenization using zirconium oxide beads in a bullet blender homogenizer (Bullet Blender Storm BBY24M, Next Advance Inc., Averill Park, NY, USA). Homogenates were centrifuged at 3,000 × g for 10 minutes; supernatant was collected and cleaned using the Amicon Ultra-0.5 centrifugal filter devices with 3K cutoff (Merck Millipore, Carrigtwohill, Ireland). Protein concentration of each eluate was measured by standard bicinchoninic acid (BCA) Protein Assay Kit (Pierce, Rockford, IL) as per manufacturer’s instruction. Following protein measurements, retinal protein samples from respective groups were pooled equally into three biological replicates, represented by RI, R2 and R3, and subsequently subjected to first dimensional gel electrophoresis.

### Sample preparation and 1-dimensional gel electrophoresis (1DE)

From each replicate, 50μg protein was transferred into 1 x LDS sample buffer (NuPAGE, Thermo Fisher) and heated at 80 ˚C for 15min under reducing condition. The samples were then separated on precast NuPAGE 8% Bis-Tris gels (Invitrogen, Karlsruhe, Germany) with 2-[N-morpholino]ethanesulfonic acid (MES) running buffer at a constant voltage of 180V at 4 °C for 30 minutes. SeeBlue Plus 2 (Invitrogen, Karlsruhe, Germany) was used as a molecular mass marker. The gels were then stained using Colloidal Blue Staining Kit (Invitrogen, Karlsruhe, Germany). Protein lanes were sliced into 25 bands per replicate and destained, reduced, alkylated dehydrated before in-gel trypsin digestion. In brief, mixture of 1: 2 (vol/vol) of 100mM NH_4_HCO_3_ and acetonitrile was used to remove the staining, 10mM 1,4-Dithiothreitol (DTT) in 100mM ammonium bicarbonate was to disulfide bonds and accordingly 55mM iodoacetamide (IAA) in 100mM NH_4_HCO_3_ for alkylation, lastly pure acetonitrile was added for gel dehydration prior to in-gel digestion with sequence grade-modified trypsin (Promega, Madison, USA) at 37˚C overnight. The peptides were then extracted firstly with acetonitrile and then with a mixture of 5% formic acid and acetonitrile 1: 2 (vol/vol), the supernatant was pooled. The extracted peptides were further purified by SOLAμ SPE Plates and Cartridges (Thermo Scientific Inc., Waltham, MA, USA) following manufacture’s instruction. The eluate was dried in SpeedVac and dissolved in 10μl of 0.1% trifluoroacetic acid (TFA) for LC-MS/MS analysis.

### Liquid chromatography (LC) - Electrospray Ionization (ESI) - MS/MS

The LC-ESI-LTQ-Orbitrap MS system is well-established in our laboratory, as described elsewhere[[25](#_ENREF_25), [26](#_ENREF_26)].Peptide fractionation was conducted in the liquid chromatography (LC) system, which contains Rheos Allegro pump (Thermo scientific, Rockford, USA) paired with an HTS PAL autosampler (CTC Analytics AG, Zwingen, Switzerland).

The system is equipped with a 30×0.5mm BioBasic C18 precolumn (Thermo Scientific, Rockford, USA) connected to a 150×0.5mm BioBasic C18 analytical column (Thermo Scientific, Rockford, USA), which has reversible hydrophobic interactions with the peptides.

The aqueous solvent A is LC-MS grade water with 0.1% (v/v) formic acid, the organic solvent B is LC-MS grade acetonitrile with 0.1% (v/v). The gradient had a running time of 60minutes per gel band, as follows; 0-35 min: 15-40 % B, 35-40 min: 40-60 % B, 40-45 min: 60-90 % B, 45-50 min: 90 % B, 50-53 min: 90-10 % B: 53-60 min: 10 % B.

The continuum MS data is collected by an ESI-LTQ Orbitrap XL-MS system (Thermo Scientific, Bremen, Germany) collects The general parameters of the instrument were set as follow: positive ion electrospray ionization mode with a spray voltage of 2.15KV and a heated capillary temperature of 220°C. Data was obtained in automatic dependent mode, an automatic acquisition switching between Orbitrap-MS and LTQ MS/MS.

The Orbitrap resolution was 30000 at m/z 400 with full scan MS spectra. Target automatic gain control (AGC) was at 1.0×10^6^ ion. Internal recalibration using polydimethlycyclosiloxane (PCM) at m/z 445.120025 ions in real time and the lock mass option was enabled in MS mode[[27](#_ENREF_27)]. Top five most intense precursor ions were obtained as tandem data and further subjected for fragmentation by collision-induced dissociation (CID). The normalized collision energy (NCE) was set to 35% with 30ms activation time and repeat count of three with 600s dynamic exclusion duration. The LTQ collected the resulting fragmented ions.

MaxQuant computational proteomics platform version 1.6.1.0 and built-in Andromeda search engine was utilized to analyze the obtained continuum MS spectra.

The tandem MS spectra were searched against UniProt database (Homo sapiens; date: Rattus?date) employing the following standard settings: Peptide mass tolerance of ±30 ppm, fxed modifcations set to carbamidomethylation of cysteine, variable modifcations assigned to oxidation of methionine and acetylation of N-termini, fragment mass tolerance set to ±0.5Da with ≥6 amino acid residues and only ‘unique plus razor peptides’ that belong to a protein were chosen, trypsin as enzyme and

maximum number of missed cleavages sites set to 2.

A target-decoy-based false discovery rate (FDR) was set to 0.01 for identification of peptide and protein. (The detailed MaxQuant parameters can be found in Suppl. file 1 “MaxQuant parameters”.)
